# Relationship Between Oral and Hand Hygiene Behaviours Among Brazilian Adolescents: Analysis of the National Adolescent School‐Based Health Survey (PeNSE 2019)

**DOI:** 10.1111/idh.70038

**Published:** 2026-03-09

**Authors:** Pedro Augusto Fernandes, Matheus França Perazzo, Maria do Carmo Matias Freire

**Affiliations:** ^1^ Postgraduate Programme, Faculty of Dentistry Federal University of Goiás Goiânia Brazil

**Keywords:** adolescents, hand disinfection, oral hygiene, students, toothbrushing

## Abstract

**Objectives:**

To investigate the association between daily toothbrushing and hand washing practices among Brazilian adolescent students.

**Methods:**

This cross‐sectional study analysed data from the 2019 Brazilian National School‐Based Health Survey. The sample comprised 120,054 schoolchildren aged 13–17 who completed an electronic questionnaire at school. The outcome variable was ‘low toothbrushing frequency’ (< twice/day). The independent variables were hand washing frequency before eating, after using the toilet, and with soap. The covariates were the adolescents' sociodemographic characteristics: sex, age, colour/race and their mother's level of education. Data analysis included Chi‐square tests with Rao‐Scott correction and logistic regression for complex samples.

**Results:**

After adjustments, adolescents who rarely/sometimes and those who never washed their hands before eating were more likely to have a low brushing frequency (< twice/day) than those who most of the time/always washed their hands before eating. Similar results were found for hand washing after using the toilet and for washing with soap.

**Conclusions:**

Associations were found between oral and hand hygiene practices. Regardless of their sociodemographic characteristics, adolescents who reported infrequent hand washing were more likely to brush their teeth less frequently. These findings reinforce the importance of adopting a common risk factors approach to oral and general health in school health programmes.

## 1 Introduction

Adolescence is a transition period between childhood and adulthood marked by the transformation of individuals, including personal, psychological, and biological changes that are noticeable to the individual and society [1]. This period encourages individual development and the adoption of health‐related behaviours, which tend to be perpetuated throughout life. Personal hygiene, such as bathing, hand washing, and toothbrushing, is among the behaviours that may influence adolescents' health.

Hand washing is an important public health measure due to its effectiveness in reducing the incidence of infectious diseases. It is one of the main general hygiene habits for preventing diarrhoea and respiratory infections [2]. The relevance of washing hands with soap became evident in the recent context of the COVID‐19 pandemic [3].

Toothbrushing is a personal hygiene measure for preventing dental caries and periodontal diseases, the most prevalent oral diseases globally [4]. Plaque accumulation and inadequate oral hygiene are crucial risk factors for periodontal diseases. Brushing teeth daily with fluoridated toothpaste is important for preventing tooth decay, especially when combined with other measures, such as reducing sugar consumption and access to fluoridated water [5]. The current recommended frequency worldwide is twice daily, mainly in the morning and the night before sleep [6].

Schools play an important role in promoting healthy behaviours among children and adolescents, including personal hygiene [7, 8]. Although toothbrushing is a component of personal hygiene, the traditional approach in health education often treats it as isolated from general health, reducing its effectiveness. When adopting a perspective of common or simultaneous risk factors for oral and general health, hygiene emerges as a shared risk factor [9]. Therefore, the relationship between hand hygiene and toothbrushing has crucial implications for implementing health promotion programmes in the school environment. Actions aimed at promoting body hygiene can positively influence students' oral hygiene and vice versa, maximising the efforts of school health teams.

A few studies among adolescent students have investigated the relationship between toothbrushing frequency and hand washing before a meal, after a meal, after using the toilet, and using soap. Results showed that adolescents who reported hand washing more frequently had a higher toothbrushing frequency than those with a lower hand washing frequency [10, 11, 12, 13, 14]. None of them simultaneously included all hand washing variables, and two were based solely on bivariate analysis. Research from Latin America is scarce [12], and evidence about adolescent students in Brazil is lacking.

Thus, the objective of the present study was to investigate the association between daily toothbrushing and hand washing practices among Brazilian adolescent schoolchildren, using data from the most recent National School‐Based Health Survey PeNSE in 2019 [15]. Based on previous studies in other countries, we hypothesised that adolescents who washed their hands less often had a lower frequency of toothbrushing. The results can be important for planning and evaluating school health promotion programmes seeking to integrate oral health into general health.

## 2 Study Population and Methodology

This cross‐sectional study analysed data from the latest National School‐Based Health Survey (PeNSE) in 2019 [15]. The PeNSE is periodically conducted by the Brazilian Institute of Geography and Statistics (IBGE) in partnership with the Ministry of Health and with the support of the Ministry of Education. The objective is to investigate the risk and protective factors for diseases among adolescent students in public and private schools. The PeNSE microdata in the public domain can be accessed at the IBGE website (http://www.ibge.gov.br).

Participants were 125,123 students aged 13–17 attending the 7th year of elementary school to the 3rd year of high school from approximately 4253 schools in 1288 cities [15]. As the study had a complex sample design, the calculated sample size was adjusted to account for the cluster effect. The stratification of schools was based on their geographical location and administrative dependence (public or private). Geographical stratification considered whether schools were located in the capital city of each of the 26 states and the Federal District, or in municipalities outside the capital city. Thus, a total of 53 geographic strata were obtained, two strata for each state and one for the Federal District. The schools were organised according to administrative dependence for each geographic stratum, totaling 106 sizing strata. The sampling plan was defined as a two‐stage cluster sample, with schools corresponding to the first stage of selection and classes of enrolled students aged 13–17 corresponding to the second. Only schools with 20 or more students of the target age group were selected. The total number of students in the selected classes formed the student sample [15].

The research protocol was approved by the Brazilian National Research Ethics Commission (Conep Report No. 3.249.268/2019). Students participated after completing a Free and Informed Consent Form. No parental consent was required. Those who agreed to participate responded to an electronic questionnaire with closed questions during class hours at their schools. They used a mobile electronic device, corresponding to a smartphone containing the self‐reported questionnaire [15]. More information on the 2019 PeNSE methodology is published elsewhere [15].

The frequency of toothbrushing and hand washing was the only variable related to personal hygiene in the 2019 PeNSE questionnaire. The present study's outcome was the adolescents' daily toothbrushing frequency, obtained through the question ‘How many times a day do you brush your teeth? (Less than once/One/Two/Three/Four or more times a day)’. For the statistical analysis, these were dichotomised into Low (less than twice) and High (twice or more), according to the current international recommendation. The three independent variables were the hand washing frequencies. The questions were ‘How often do you wash your hands (before eating/after using the bathroom/with soap)?’ and the response options were Never/Rarely/Sometimes/Most of the time/Always.

The covariates were the adolescents' sociodemographic characteristics with the potential to influence the outcome: sex (Male/Female), age (13–15/16–17 years), colour/race, and their mother's level of education. The original five categories of colour/race were maintained to comply with the Brazilian classification: white, brown, black, yellow (Asian descendants) and indigenous. The mother's education level was dichotomised according to the national educational system: Low (did not study/incomplete elementary school) and High (completed elementary school to completed higher education).

Descriptive statistics were initially performed to estimate the frequencies of the variables. Bivariate analyses to identify variables associated with the outcome ‘low daily toothbrushing frequency’ (less than twice) were performed using the Rao‐Scott chi‐square test, with a significance at *p* < 0.05. In the next step, logistic regression analysis was used, considering the Odds Ratio (OR) estimates and their respective 95% confidence intervals (CI). To facilitate interpretations, hand washing frequencies were grouped into three categories: Never, Rarely/Sometimes, and Most of the time/Always. Reference categories were more favourable indicators of behaviours and sociodemographic characteristics. All variables associated with the outcome in the Rao‐Scott chi‐square test were selected for the unadjusted analysis. For the adjusted regression, those that remained significant (*p* < 0.05) in the unadjusted stage were included. Each of the three independent variables was analysed individually, and three regression models were constructed, adjusting for the sociodemographic characteristics of the respondents (Models 1, 2 and 3).

The complex sampling design used in the 2019 PeNSE was considered in the statistical procedures using weighting and specialised procedures available in the SPSS statistical software Version 28 (IBM Corporation, Armonk, NY, USA).

## 3 Results

Of the 125,123 students in the PeNSE data bank, those with non‐response in the variables selected for this study were excluded, resulting in 120,054 respondents (Table 1). The majority were female (51.1%), aged between 13 and 15 (66%). The most frequent colour/race was brown (43.6%). Approximately 42% of the adolescents' mothers had a high level of education.

**TABLE 1:** Frequency distribution and bivariate analysis between the adolescents' daily toothbrushing frequency and independent variables.

Independent variables	Daily toothbrushing frequency (%)			
	%	Low (< twice) 5.7%	High (twice or more) 94.3%	*p* *
Sex				< 0.001
Male	48.9	6.7	93.3	
Female	51.1	4.7	95.3	
Age				0.020
13–15	66.0	5.9	94.1	
16–17	34.0	5.3	94.7	
Colour/Race				0.002
Brown	43.6	5.6	94.4	
White	39.0	5.5	94.5	
Black	11.0	5.5	94.5	
Yellow	3.6	8.7	91.3	
Indigenous	2.8	6.8	93.2	
Mother's education level				0.003
Low	17.1	6.2	93.8	
High	82.9	5.2	94.8	
Don't know	15.5			
Frequency of daily toothbrushing				
Less than once	0.7			
Once	4.7			
Twice	27.5			
Three times	43.8			
Four times or more	23.3			
Frequency of hand washing before eating				< 0.001
Never	2.8	25.3	74.7	
Rarely	12.0	11.9	88.1	
Sometimes	20.5	7.0	93.0	
Most of the time	27.6	4.4	95.6	
Always	37.1	2.9	97.1	
Frequency of hand washing after using the toilet				< 0.001
Never	1.3	31.4	68.6	
Rarely	4.6	16.6	83.4	
Sometimes	9.1	10.1	89.9	
Most of the time	17.3	6.0	94.0	
Always	67.7	3.9	96.1	
Frequency of hand washing with soap				< 0.001
Never	1.3	29.7	70.3	
Rarely	6.3	14.1	85.9	
Sometimes	14.0	8.7	91.3	
Most of the time	25.0	4.9	95.1	
Always	53.4	3.5	96.5	

*Note:* Brazilian National School‐based Health Survey (PeNSE) 2019.

* Chi‐square with Rao‐Scott correction for complex samples.

The descriptive results of the adolescents' hygiene behaviours are in Table 1. The most cited toothbrushing frequency was 3 times per day (43.8%), while 0.7% of the sample reported not brushing daily. Regarding hand washing, there was a predominance of the ‘Always’ category in the three variables investigated: 37.1% before eating, 67.7% after using the toilet and 53.4% washing with soap.

The prevalence of the outcome “low daily toothbrushing frequency” (less than twice) was 5.7% (95% CI = 5.4–6.0). The Rao‐Scott test indicated significant associations between the outcome and each of the three variables related to hand washing (*p* < 0.001) (Table 1). Lower proportions of adolescents with infrequent toothbrushing were observed as the frequency of hand washing increased. Significant bivariate associations were also found between the outcome and each of the sociodemographic characteristics.

Table 2 shows the results of the logistic regression analysis. In the unadjusted model, the three independent variables, as well as the sociodemographic characteristics of the students, were associated with the outcome ‘low daily toothbrushing frequency’ (*p* < 0.05). After adjusting for the sociodemographic characteristics of the respondents, all associations remained.

**TABLE 2:** Logistic regression analysis of the association between the adolescents' daily toothbrushing and hand washing frequency.

	Outcome: Low daily tooth brushing frequency (twice or more)							
	Unadjusted^a^ OR (95% CI)	*p*	Adjusted model 1^b^ OR (95% CI) frequency of hand washing before eating	*p*	Adjusted model 2^b^ OR (95% CI) frequency of hand washing after using the toilet	*p*	Adjusted model 3^b^ OR (95% CI) frequency of hand washing with soap	*p*
Frequency of hand washing before eating		< 0.001		< 0.001				
Never	9.37 (7.76–11.32)		10.14 (8.20–12.53)					
Rarely/Sometimes	2.67 (2.38–2.99)		2.88 (2.54–3.26)					
Most of the time/Always	1		1					
Frequency of hand washing after using the toilet		< 0.001				< 0.001		
Never	10.28 (8.18–12.93)				11.02 (8.56–14.19)			
Rarely/Sometimes	3.13 (2.77–3.54)				3.21 (2.81–3.67)			
Most of the time/Always	1				1			
Frequency of hand washing with soap		< 0.001						< 0.001
Never	10.39 (8.35–12.93)						12.37 (9.69–15.80)	
Rarely/Sometimes	2.86 (2.55–3.22)						2.79 (2.45–3.16)	
Most of the time/Always	1						1	
Sex		< 0.001		< 0.001		< 0.001		< 0.001
Male	1.46 (1.31–1.62)		1.54 (1.37–1.73)		1.38 (1.22–1.56)		1.36 (1.21–1.54)	
Female	1		1		1		1	
Age		0.020		0.025		0.103		0.110
13–15	1.14 (1.02–1.27)		1.15 (1.02–1.30)		1.11 (0.98–1.25)		1.11 (0.98–1.25)	
16–17	1		1		1		1	
Colour/Race		0.005		0.020		0.019		0.010
Brown	1.04 (0.92–1.17)		1.06 (0.92–1.21)		1.00 (0.88–1.15)		1.01 (0.88–1.15)	
Black	1.00 (0.85–1.19)		0.95 (0.79–1.15)		0.91 (0.75–1.10)		0.90 (0.74–1.09)	
Yellow	1.66 (1.26–2.19)		1.69 (1.22–2.36)		1.67 (1.20–2.33)		1.72 (1.24–2.39)	
Indigenous	1.26 (0.92–1.73)		1.18 (0.84–1.66)		1.05 (0.75–1.48)		1.09 (0.77–1.53)	
White	1		1		1		1	
Mother's education level		0.003		< 0.001		0.021		0.033
Low	1.21 (1.07–1.37)		1.26 (1.11–1.44)		1.16 (1.02–1.32)		1.15 (1.01–1.31)	
High	1		1		1		1	

*Note:* Brazilian National School‐based Health Survey (PeNSE) 2019. *N* = 120,054. All calculations were performed for complex samples.

Abbreviations: 95% CI, 95% confidence interval; OR, odds ratio.

^a^ Variables with a *p* < 0.25 in the Rao‐Scott test.

^b^ Model adjusted for all variables with a *p* < 0.05 in the unadjusted regression analysis.

Compared to adolescents who washed their hands before eating most of the time/always, those who rarely/sometimes washed were 2.88 times more likely, and those who never washed their hands were 10.14 times more likely to have low toothbrushing frequency (Model 1). Regarding hand washing after using the toilet, adolescents who rarely/sometimes washed were 3.21 times more likely, and those who never washed were 11.02 times more likely to have low toothbrushing frequency than adolescents who washed their hands most of the time/always (Model 2). Adolescents who rarely/sometimes used soap were 2.79 times more likely, and those who never used it were 12.37 times more likely to have low toothbrushing frequency than those who used soap most of the time/always (Model 3).

In each model, all covariates were associated with the outcome (Table 2). Higher odds of low toothbrushing frequency were found among male adolescents, younger (13–15 years old), Asian and whose mothers had lower education (*p* < 0.05).

## 4 Discussion

The present study investigated the relationship between hand washing and oral hygiene among Brazilian adolescent students. The hypothesis that those who washed their hands less often had a lower daily frequency of toothbrushing was confirmed. The findings suggest that these are both components of broader personal hygiene behaviours. Statistical associations were found in the bivariate and multiple regression analyses, indicating the consistency of the findings. Also, the associations between the hygiene variables were regardless of the adolescents' sociodemographic characteristics.

Our results corroborate those reported in other countries [10, 11, 12, 13, 14], adding knowledge to the existing body of evidence. Research on this topic in Latin America and the Caribbean [12] is restricted to countries participating in the Global School‐based Student Health Survey (GSHS) and does not include Brazil. This is also the only study to investigate three indicators of hand washing.

The present study has implications for the school environment, which is an important setting for promoting adolescent health [7, 8]. Traditionally, school‐based oral health promotion has been carried out separately from general health promotion. Specifically, strategies for promoting oral hygiene are not usually implemented jointly by dental staff and other healthcare professionals. However, the link between hand washing and toothbrushing frequencies suggests that interventions aimed at improving oral hygiene may also impact body hygiene practices, and vice versa. It may also be possible to estimate the risk of poor oral hygiene based on low hand washing frequency. These findings highlight the importance of addressing common risk factors, as poor hygiene can contribute to oral diseases and other systemic conditions [9]. Therefore, the implementation of comprehensive health promotion strategies is necessary in schools, strengthening the joint approach to oral and general health. Health education strategies, such as encouraging hand washing with soap before toothbrushing with fluoridated toothpaste after school meals, could have a positive effect. However, these strategies require access to personal hygiene products, water and sanitation facilities, as well as adequate training for school staff.

The high frequency of hygiene behaviours among Brazilian adolescents also deserves attention, considering their potential impact on health status, quality of life and school performance. The high prevalence of toothbrushing twice or more a day (94.6%) is higher than that reported in other low and middle‐income countries [12, 16, 17] and European countries [18]. Similarly, a high proportion of adolescents participating in this study reported that they washed their hands most of the time or always, mainly after using the toilet (84.0%), contrasting with other countries [12, 13, 16, 19]. Differences in personal hygiene among countries may be explained by varying cultural values, religious issues, socioeconomic factors, and access to resources. Wealthier nations generally have higher standards and accessibility to hygiene facilities, while developing countries often face challenges with clean water and infrastructure, impacting daily hygiene practices [20]. In Brazil, which is considered an upper‐middle‐income country, personal hygiene is an important part of routine, reflecting its culture and traditions.

Despite the high prevalence of handwashing, several adolescents in the present study reported poor hygiene practices (e.g., 0.7% did not brush their teeth daily, and 2.8% never washed their hands before meals). These results highlight the importance of monitoring risk and protective factors among adolescent students, as well as the need for targeted health education for those at higher risk.

The main strength of this study is its large, nationally representative, school‐based randomised sample of adolescent schoolchildren, which may provide good external validity. The PeNSE also used a standardised, validated questionnaire to assess hygiene practices using three indicators of hand washing to enable a more comprehensive analysis. As this national school survey is carried out systematically in Brazil, it is recommended that future longitudinal studies monitor whether the found associations change over time. Some limitations inherent to cross‐sectional studies and the use of secondary data should be considered when interpreting the present results. Using the PeNSE database prevents analysis of the consistency and accuracy of responses. Also, data were collected through a self‐administered questionnaire, which is subject to a lack of precision mainly due to participants' difficulties in understanding the questions and memory bias.

Our results may be relevant to the evaluation and evidence‐based planning of the School Health Programme in Brazil [21] and similar school health interventions globally. For a broader perspective on adolescents' hygiene behaviours, we recommend that future editions of the PeNSE [15] and other school surveys include personal hygiene behaviours, such as bathing and hair washing, which were investigated in previous studies [10, 11].

## 5 Conclusion

Significant associations were found between oral and hand hygiene practices among Brazilian adolescent schoolchildren. Regardless of their sociodemographic characteristics, adolescents who reported infrequent hand washing were more likely to brush their teeth less frequently. These findings have implications for dental practice and health promotion in schools. They also indicate the importance of adopting a common risk factors approach to oral and general health because poor hygiene may contribute to oral diseases and other systemic conditions.

## 6 Clinical Relevance

### 6.1 Scientific Rationale for Study

The relationship between oral and hand hygiene among adolescents is crucial for planning effective school health programmes; however, it is poorly described in Latin America.

### 6.2 Principal Findings

Adolescent students who reported infrequent hand washing were more likely to brush their teeth less frequently, regardless of their sociodemographic characteristics.

### 6.3 Practical Implications

The link between hand washing and toothbrushing frequencies suggests that interventions targeting oral hygiene may also impact body hygiene practices and vice versa.

## Author Contributions

M.C.M.F. and M.F.P. conceived the ideas; P.A.F. collected the data; P.A.F., M.C.M.F. and M.F.P. analysed the data; and P.A.F. and M.C.M.F. led the writing.

## Funding

This work was supported by Coordenação de Aperfeiçoamento de Pessoal de Nível Superior, 001.

## Conflicts of Interest

The authors declare no conflicts of interest.

## Data Availability

Data available on request from the authors.
